# A Multiwell Platform for Studying Stiffness-Dependent Cell Biology

**DOI:** 10.1371/journal.pone.0019929

**Published:** 2011-05-27

**Authors:** Justin D. Mih, Asma S. Sharif, Fei Liu, Aleksandar Marinkovic, Matthew M. Symer, Daniel J. Tschumperlin

**Affiliations:** Molecular and Integrative Physiological Sciences, Department of Environmental Health, Harvard School of Public Health, Boston, Massachusetts, United States of America; The University of Akron, United States of America

## Abstract

Adherent cells are typically cultured on rigid substrates that are orders of magnitude stiffer than their tissue of origin. Here, we describe a method to rapidly fabricate 96 and 384 well platforms for routine screening of cells in tissue-relevant stiffness contexts. Briefly, polyacrylamide (PA) hydrogels are cast in glass-bottom plates, functionalized with collagen, and sterilized for cell culture. The Young's modulus of each substrate can be specified from 0.3 to 55 kPa, with collagen surface density held constant over the stiffness range. Using automated fluorescence microscopy, we captured the morphological variations of 7 cell types cultured across a physiological range of stiffness within a 384 well plate. We performed assays of cell number, proliferation, and apoptosis in 96 wells and resolved distinct profiles of cell growth as a function of stiffness among primary and immortalized cell lines. We found that the stiffness-dependent growth of normal human lung fibroblasts is largely invariant with collagen density, and that differences in their accumulation are amplified by increasing serum concentration. Further, we performed a screen of 18 bioactive small molecules and identified compounds with enhanced or reduced effects on soft versus rigid substrates, including blebbistatin, which abolished the suppression of lung fibroblast growth at 1 kPa. The ability to deploy PA gels in multiwell plates for high throughput analysis of cells in tissue-relevant environments opens new opportunities for the discovery of cellular responses that operate in specific stiffness regimes.

## Introduction

The stiffness of the extracellular matrix is a vital physical cue that regulates cellular fate and function [1]. For instance, self-renewal and lineage commitment of stem cells both vary with the stiffness of the underlying substrate, while the differentiated function of myoblasts and cardiomyocytes depend on optimal substrate stiffness [2]–[5]. The relevance of substrate stiffness across many biological settings has major implications for cell and biomaterials research, particularly because it is a parameter that can be controlled *in vitro*. Yet, for all its potential, the study of cells on substrates replicating tissue stiffness has not been reconciled with standard high throughput approaches, preventing a more systematic exploration of its effects.

To span the physiologic range of soft tissues, polyacrylamide (PA) is commonly the material of choice due to its broad range of linear elastic behavior. However, existing methods to fabricate PA gels for cell culture require labor-intensive production in relatively small batches. Semler et al. sought to overcome this limitation by punching cylindrical PA gels from large sheets and mounting them within multiwell plates [6], but this method remains tedious and in our hands was not compatible with soft PA gels. More recently, a number of microfabrication approaches have been advanced to study cells in microwells or on top of flexible post arrays [7], [8]. While offering unique opportunities for dissecting stiffness-dependent effects, these tools require specialized procedures for manufacturing and data analysis that are not immediately accessible to many laboratories.

We decided to revisit the original study describing in 1997 the culture of cells on stiffness-tunable PA gels [9], and surmised that scaling the procedure to a multiwell format could be achieved with minor modifications. Subsequently, we arrived at a method to rapidly cast PA gels in 96 and 384 well plates, and used this platform to culture a diverse set of cell types across a physiological stiffness range, detect differences in their accumulation, and gauge the interactive effects of substrate stiffness with soluble and insoluble cues. Finally, we performed a small-scale, pharmacological screen of cells cultured on soft and rigid substrates, and identified drug responses that are highly contingent upon the stiffness context.

## Results

### Characterization of stiffness-controlled multiwell plates

**Figure 1:**
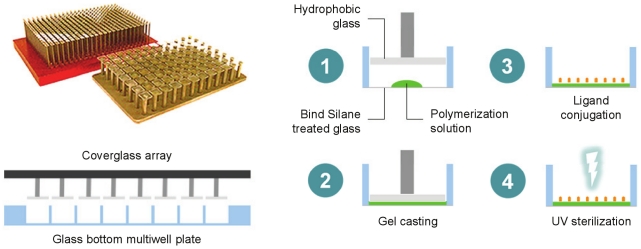
Schematic of polyacrylamide gel incorporation into a multiwell plate. PA gels are cast using an array of coverglass to sandwich polymerization solutions within a multiwell plate, followed by ligand conjugation and sterilization.

**Figure 2:**
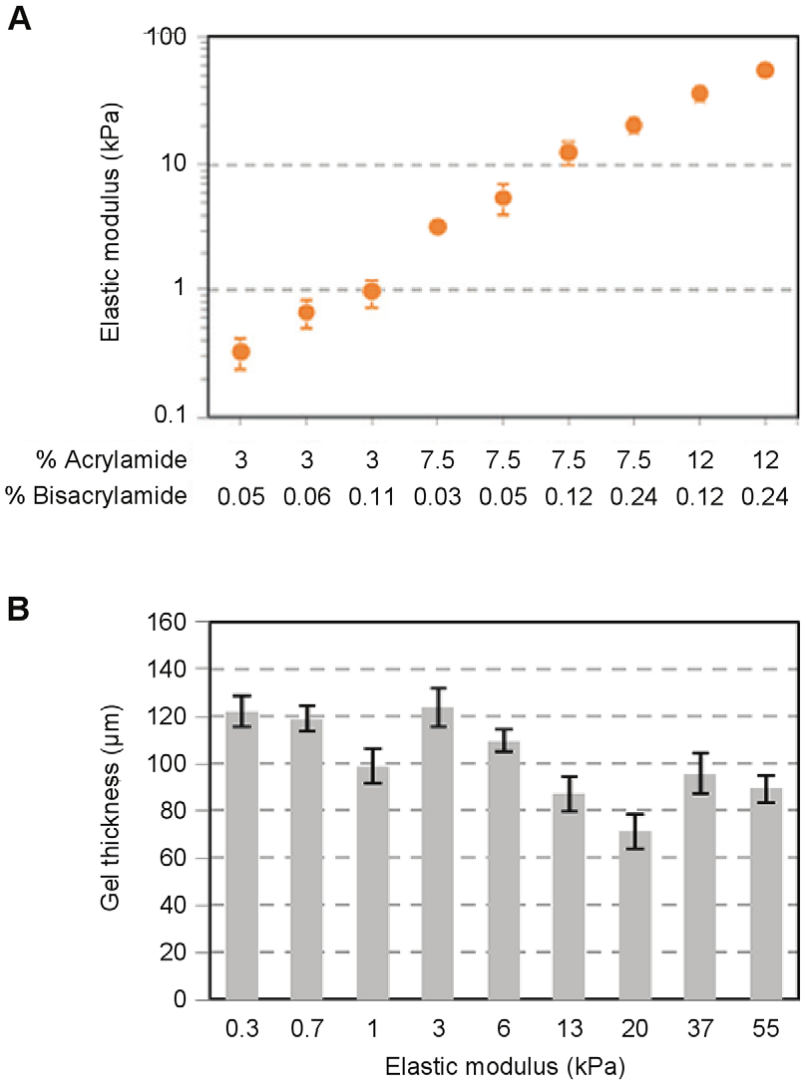
Measurement of substrate elastic modulus and thickness. (A) Acrylamide: bisacrylamide content was extrapolated from Yeung et al. [10] to target a broad physiologically relevant stiffness range. Young's modulus was determined by AFM microindentation of gels cast within three separate 96 well plates. Data are mean ± SD (n = 3). (B) Final gel thicknesses are within 11% CV for each stiffness condition, but variable between stiffness due to differences in gel swelling with bisacrylamide crosslink content. Data are mean ± SD (n = 5) from one 96 well plate.

**Figure 3:**
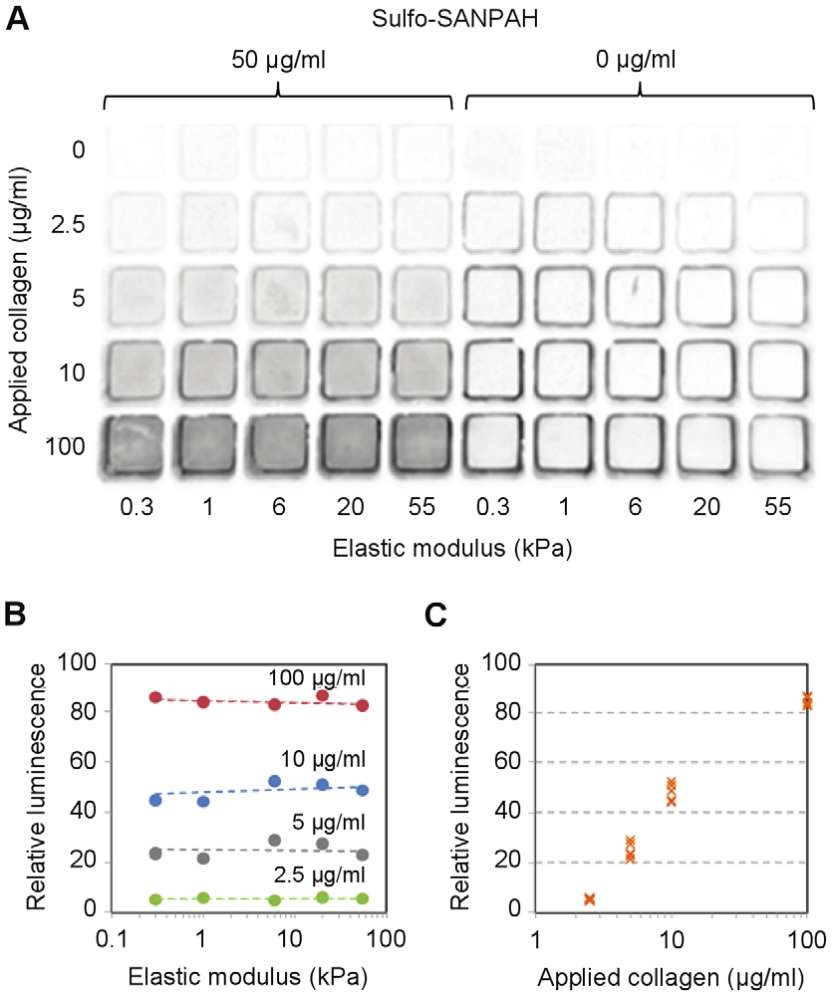
Surface density of collagen across matrix stiffness. (A) Chemiluminescence-based detection of gel-bound collagen. The no sulfo-SANPAH controls assess non-specific adsorption of collagen to the gels. (B) Average pixel intensities of the chemiluminescent signals imaged in (A). (C) Relationship between applied collagen concentration and signal intensity measured across all stiffness conditions in (B).

PA gels were cast in 96 and 384 well plates (**Fig. 1**) as described in Materials and Methods. The elastic moduli of substrates formed by 9 different concentrations of acrylamide:bisacrylamide ranged from 0.3 to 55 kPa, as measured by AFM microindentation (**Fig. 2A**). For any given stiffness, gel thickness was relatively constant among wells (<11% CV), but varied from 70–120 µm across the stiffness range (**Fig. 2B**). The observed differences in gel thickness are reflective of increased swelling with reduced bisacrylamide crosslinking at a given concentration of acrylamide [11]. For all gels >1 kPa, within-well variations in thickness were negligible; for extremely soft gels (0.3 and 1 kPa), within-well thickness did not vary by more than 5%. Slight distortions in gel uniformity did occur within 0.1 mm of the polystyrene walls of each well, and these distortions were not considered for analysis of gel thickness. The density of gel-bound collagen, which was adjustable over a 12-fold range, could be tuned independently of stiffness (**Fig. 3A**–**C**). Overall, the gels were highly uniform and the subtle variations in thickness did not interfere with microscopy or cell-based assays. All substrates remained firmly bound to the plate through vigorous washing, media changes, and cell culture conditions.

### Automated fluorescent imaging in a 384 well plate

**Figure 4:**
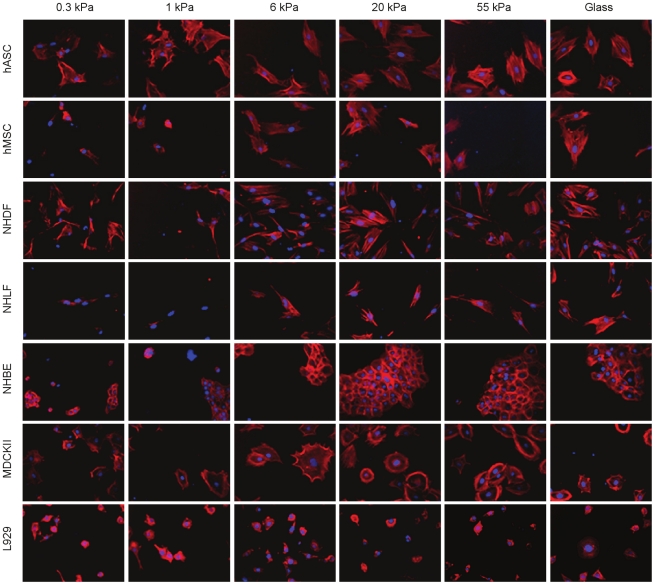
Automated imaging of cell morphology in a 384 well plate. Seven cell types (**Table 1**) cultured across increasing substrate stiffness, stained for F-actin (red) and nuclei (blue). Images were obtained at 200X magnification.

To demonstrate compatibility with automated imaging systems, we fabricated PA gels spanning the entire stiffness range (0.3–55 kPa) in a 384 well plate and seeded 7 cell types at various densities. After 24 hours in culture, the cells were fixed and stained to visualize f-actin and nuclei. Using autofocusing, we captured images of cells on all substrates, including glass (**Fig. 4**). Prominent morphological transitions in the 1–6 kPa range were evident in many of the primary and immortalized cell lines, with the exception of L929 cells, which were virtually indistinguishable under all stiffness conditions.

### Profiling cell growth patterns across substrate stiffness

**Figure 5:**
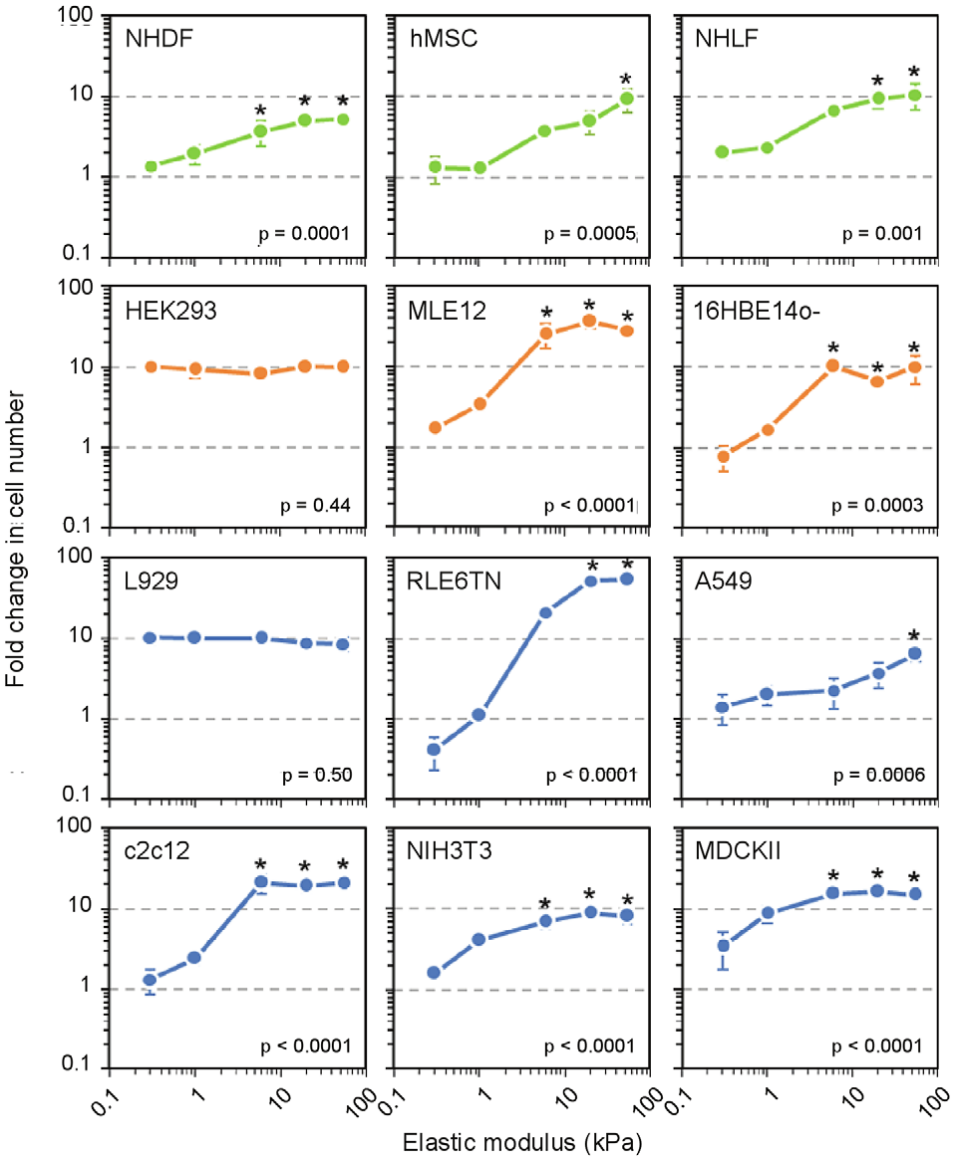
Stiffness-dependent growth profiles. 12 cell types (**Table 1**) cultured across 5 stiffness conditions in 96 well plates. Fold change represents number of attached cells at 72 h versus the 4 h attachment density. Cells are classified as normal (green), SV40- or adenovirus-transformed (orange), or spontaneously immortalized (blue). Error bars indicate mean ± SD (n = 3). P values from one-way ANOVA are indicated in each plot. *p<0.05 vs. growth on the softest substrate (0.3 kPa) by Tukey's test.

Various cell types have been reported to grow more rapidly as substrate stiffness increases [12]–[17]. To explore this phenomenon systematically, we surveyed the effect of substrate stiffness on the accumulation (number of attached cells at 72 hours versus 4 hours post-seeding) of 12 cell types (**Fig. 5** and **Table 1**). Cells were seeded at a subconfluent density (<50 cells/mm^2^) and cultured in 10% serum on 5 elastic moduli ranging from 0.3 to 55 kPa. We categorized the cell types as normal (NHLF, NHDF, hMSC), SV40- or adenovirus-transformed (HEK293, MLE12, 16HBE14o-), or spontaneously immortalized (L929, NIH3T3, c2c12, MDCKII, RLE6TN, A549). After 72 hours, a majority of cell types exhibited generally increasing accumulation across stiffness. Strikingly, the growth of L929 and HEK293 cells was completely insensitive to stiffness, supporting the idea that the ability to detect or respond to mechanical signals may be lost in some cell types that have undergone malignant transformation [13], [17].

**Table 1:** Cell types.

16HBE14o-	Human bronchial epithelial cell line
A549	Human lung adenocarcinoma cell line
c2c12	Mouse myoblast cell line
hASC	Human adipose-derived stem cells
HEK293	Human embryonic kidney cell line
hMSC	Human bone marrow-derived stem cells
L929	Mouse fibroblast cell line
MDCKII	Madin Darby canine kidney epithelial cell line
MLE12	Mouse lung epithelial cell line
NHBE	Normal human bronchial epithelial cells
NHDF	Normal human dermal fibroblasts
NHLF	Normal human lung fibroblasts
NIH3T3	Mouse embryonic fibroblast cell line
RLE6TN	Rat lung epithelial cell line

### Substrate stiffness interactions with ligand density and soluble factors

**Figure 6:**
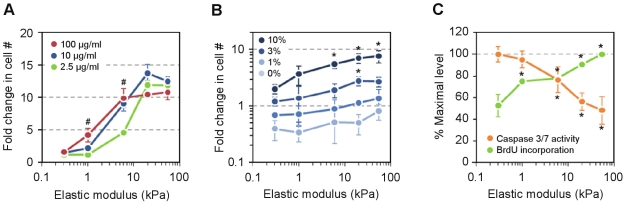
Substrate stiffness interactions with soluble and matrix factors. (A) Effect of varying collagen ligand density on cell accumulation. Applied collagen concentrations are indicated. Fold change represents number of attached cells at 72 h versus the 4 h attachment density. (B) NHLFs accumulate more rapidly on stiffer substrates in high serum concentrations. No significant differences in accumulation are detected in low serum concentrations, but both 0% and 1% conditions exhibit statistically positive trends with increasing stiffness (p = 0.0032, p = 0.013, respectively, by two-tailed t-test). (C) Increasing matrix stiffness promotes BrdU incorporation while suppressing caspase 3/7 activity in cells cultured in 10% serum. Values are normalized per cell number and expressed relative to the maximal value. Error bars indicate mean ± SD (n = 3). *p<0.05 vs. effect on the softest substrate (0.3 kPa) by Tukey's test. #p<0.05 vs. effect at the lowest collagen density by t-test.

Selecting normal human lung fibroblasts for further study, we first considered how the density of gel-bound collagen might interact with stiffness to generate the accumulation profile (**Fig. 6A**). The effect of increasing collagen up to 12-fold over a minimally detectable threshold significantly enhanced cell accumulation on 1 and 6 kPa substrates. However, regardless of ligand density, cell accumulation was similarly robust on stiffer substrates (20 and 55 kPa) and suppressed on the lowest stiffness (0.3 kPa). Thus, the overall profile of cell growth appeared largely driven by stiffness. Next, we fixed collagen at an intermediate density (10 µg/ml applied collagen) and assessed the effect of varying serum concentration (**Fig. 6B**). At 10% serum, cell accumulation was net positive across the entire stiffness range, with significantly higher accumulation on 6, 20, and 55 kPa substrates versus the 0.3 kPa substrate. The apparent promotion of cell proliferation was confirmed by the profile of BrdU incorporation across stiffness (**Fig. 6C**). Conversely, the suppression of cell accumulation at low stiffness was consistent with increased caspase 3/7 activity, indicative of apoptosis (**Fig. 6C**). Reducing the serum concentration to 3% downshifted the growth curve, and under these circumstances, only cell accumulation at 20 kPa was significantly higher than at 0.3 kPa. Upon further reduction of serum, no significant differences in cell accumulation were detected among the stiffness conditions. However, the overall relationship between stiffness and cell accumulation exhibited a significantly positive trend, suggesting that stiffer environments may enhance cell survival in growth-restrictive circumstances. These results emphasize the robust effect of increased substrate stiffness on cell accumulation through both growth promotion and apoptosis resistance across a wide range of matrix density and serum contexts.

### Screening substrate stiffness-dependent drug responses

**Figure 7:**
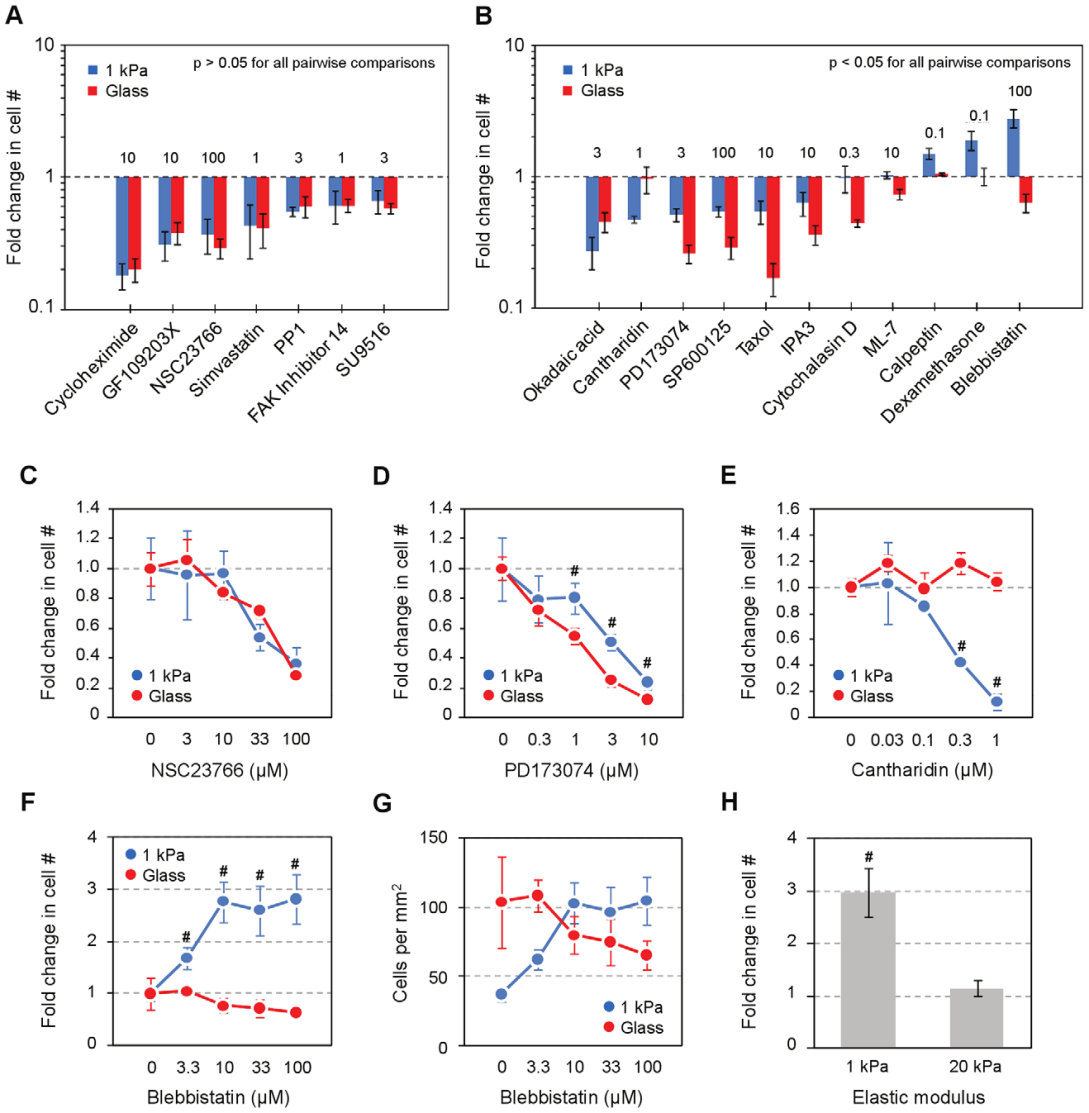
Modification of drug responses on 1 kPa vs. glass. (A) Compounds that inhibit cell growth with similar potency on 1 kPa and collagen-coated glass (rigid). For each stiffness condition, fold change represents the number of cells at the indicated dose (µM) vs. a zero dose control after 72 hours in culture. (B) Compounds that affect relative cell growth differentially (p<0.05) between the two stiffness contexts. (C) Stiffness-independent effects of a Rac1 inhibitor. (D) Reduced potency of an FGF1 receptor inhibitor on 1kPa vs. glass. (E) Increased potency of a PP1 inhibitor on 1 kPa vs. glass. (F) Stimulation of cell growth on 1 kPa by a myosin II inhibitor. (G) Absolute cell numbers following blebbistatin treatment in F. Error bars indicate mean ± SD (n = 3) in A–G. (H) Blebbistatin (10 µM) stimulates cell growth on 1 kPa substrates, with no effect on 20 kPa substrates. Fold change indicates cell number over no drug control. Error bars indicate mean ± SEM (n = 5 experiments). #p<0.05 vs. the rigid condition by t-test for all panels.

The ability to survey stiffness-specified biology efficiently enabled us to conduct a small-scale pharmacological screen of known or suspected inhibitors of cell proliferation. To increase throughput, we manufactured 96 well plates specifying only two stiffness contexts: the approximate elastic modulus of lung parenchyma (1 kPa) and rigid glass [18]. We seeded lung fibroblasts in 10% serum at a subconfluent density and exposed them to a range of drug concentrations for 72 hours. An unexpectedly diverse set of context-specific responses emerged from the evaluation of only 18 small molecules (**Fig. 7A-H** and **Table 2**). Not surprisingly, the effects of a number of compounds were indistinguishable between 1 kPa and glass (**Fig. 7A**), exemplified by NSC 23766, an inhibitor of Rac1 (**Fig. 7C**). However, the responses to a large subset of compounds subtly but significantly differed in magnitude between soft and rigid contexts (**Fig. 7B**), as was the case for PD173074, an inhibitor of FGF receptor 1 (**Fig. 7D**), and cantharidin, an inhibitor of protein phosphatases 1 and 2A (**Fig. 7E**). While the differences in growth attenuation by these compounds were relatively modest in scope, the differences were maintained across a concentration range of 10- to 33-fold, indicating that the influence of substrate stiffness on such drug responses is surprisingly robust. Notably, this small screen not only identified compounds that were superior in attenuating cell growth on soft substrates (e.g., cantharidin, okadaic acid), but also several compounds that were highly effective on rigid substrates but less so on soft substrates (e.g., PD173074, taxol, cytochalasin D), and surprisingly, one compound (blebbistatin) that was entirely growth stimulatory on soft substrates but inhibitory on glass when applied at 100 µM (**Fig. 7F**). In fact, we consistently observed that 10 µM blebbistatin was sufficient to fully rescue cell growth, which is normally suppressed at 1 kPa, to maximal levels occurring on glass (**Fig. 7G**). We confirmed the stiffness-sensitivity of this effect by comparing the responses to 10 µM blebbistatin on 1 and 20 kPa hydrogels. Across 5 independent experiments we observed a 3-fold increase in cell number on 1 kPa substrates, with no change on 20 kPa substrates, confirming the robustness of the effect and its specificity to soft substrates (**Fig. 7H**).

**Table 2:** Molecular actions of tested compounds.

Blebbistatin	Myosin II inhibitor
Calpeptin	Inhibits calpain, cathespin L, and myosin light chain phosphorylation
Cantharidin	Inhibits protein phosphatases 1 and 2A
Cycloheximide	Block tRNA binding and release from ribosomes
Cytochalasin D	Disrupts actin polymerization
Dexamethasone	Glucocorticoid and anti-inflammatory
FAK Inhibitor 14	Inhibits focal adhesion kinase
GF 109203X	Inhibits protein kinase C
IPA3	Inhibits group 1 p21-activated kinase
ML-7	Inhibits myosin light chain kinase
NSC 23766	Inhibits Rac1
Okadaic acid	Inhibits protein phosphatases 1 and 2A
PD 173074	Inhibits FGF receptor 1 and FGF receptor 3
PP1	Inhibits Src tyrosine kinase
Simvastatin	Inhibits HMG-CoA reductase
SP600125	Inhibits JNK
SU 9516	Inhibits Cdk2
Taxol	Promotes microtubule assembly and inhibits microtubule disassembly

## Discussion

Cell fate decisions in adherent cells are strongly influenced by cues provided by the physical environment, including the stiffness of the surrounding matrix. To accelerate research in this emerging area, we developed a method to specify the context of physiological tissue stiffness in a multiwell plate. The strategy described here is similar to the common practice of polymerizing PA gels between two glass substrates, only that a ‘coverglass array’ is used to cast individual gels simultaneously. Such an apparatus can be conceived of in a number of ways, but we found it sufficient to affix properly sized glass to 96 and 384 pin steel replicators.

A more formidable challenge is the polymerization of a uniform gel layer that is durably bound to the entire well surface. This was not immediately achievable following the original method to fabricate PA gels for cell culture [19] or related variant methods [20]-[23] so we modified key steps. First, to form an acrylamide-reactive surface, we pretreated glass-bottom plates with 3-methacryloxypropyltrimethoxysilane (Bind Silane), which bound PA gels more robustly than conventional treatment with NaOH/3-aminopropyltrimethoxysilane/glutaraldehyde. Similar results can be achieved using a number of vinyl-presenting silanes, such as allyltrichlorosilane [23]. These modifications increase the hydrophobicity of the glass, which is necessary to prevent the formation of a concave meniscus within the well. Second, we added sodium bisulfate, an oxygen scavenger used for defect-free preparation of precast PA gels [24], to the polymerization reaction. This eliminated any need to degas the solutions prior to each casting run and was effective in driving complete polymerization at the gel edges. We targeted the final thickness of the gels to be at least three times the thickness (∼20 µm) at which MSCs cultured on an E = 1 kPa substrate begin to respond to the rigidity of the underlying glass substrate [23]. Another potentially confounding aspect we considered was surface creasing, which can occur when a geometrically constrained gel of low modulus undergoes swelling [25]. This phenomenon should not be triggered at the 3% acrylamide concentration used for 0.3, 0.7, and 1 kPa gels [26], which we confirmed visually. Finally, following standard methods, we used a heterobifunctional crosslinker to conjugate the otherwise inert gels with monomeric collagen I, though any number of crosslinking strategies and cell attachment ligands can be employed. The resulting ligand density, visualized throughout the entire gel surface by antibody-based chemiluminescence, exhibited a high degree of uniformity across the stiffness range studied here.

Altogether, this approach makes it possible to more rapidly define the physical characteristics of matrices that promote cell differentiation and survey the effects of exogenous factors (e.g., cytokines, growth factors, small molecule therapeutics) in conjunction with matrix stiffness. As our results demonstrate, such studies may identify crucial differences in cell responses that depend on substrate stiffness, and even uncover those that are obscured altogether with assays performed on traditionally rigid tissue culture substrates.

One prominent effect of matrix stiffness that has been widely reported is the promotion of cell growth with increasing substrate stiffness. Our study of a panel of 12 cell lines and primary low passage cell types confirms the generally positive effect of increasing stiffness on cell accumulation (net change in cell number due to proliferation, death or detachment). Notable exceptions to this rule are L929 and HEK293 cells, which exhibited no relative preference for growth across the entire range of stiffness from 0.3 to 55 kPa. These findings echo earlier observations of stiffness insensitivity in H-ras transformed NIH 3T3 cells [13], but contrast with the behavior of 16HBE14o- cells, which are SV40 transformed, and A549 cells, which are derived from a human lung adenocarcinoma, suggesting complexity in the molecular mechanisms by which stiffness sensitivity is lost. This variability in matrix stiffness sensitivity has recently been exploited by Kostic et al. [27] to identify single cell clones of MDA-MB-231 breast cancer cell lines that either thrive or fail to grow on soft substrates. Further expanding on the role of matrix stiffness on tumor cell growth control, Tilghman et al. [17] recently showed that a large panel of cancer cell lines could be segregated into stiffness-dependent and independent classes. Further dissection of genetic and molecular differences among cell lines and clones exhibiting differential sensitivities to matrix stiffness may provide unique insight into mechanisms of stiffness transduction.

Among the primary cells examined, fibroblasts derived from the lung and skin, tissues which differ in stiffness by approximately 10-fold [18], [28], exhibited largely similar behaviors. The general trend in cell growth with increasing stiffness was strikingly similar to that observed in vascular smooth muscle cells, mammary epithelial cells, and mouse embryonic fibroblasts [12], suggesting that cell growth relationships with matrix stiffness are robust and surprisingly well-conserved across cells derived from multiple tissues. Our finding of greatly suppressed hMSC growth on substrates of low stiffness is also consistent with observations of Winer et al. [16], who reported that substrates with Young's modulus of 0.25 kPa provoked quiescence in hMSCs. The observed conservation among cell growth responses to substrate stiffness contrasts with observations of divergent lineage specification over unique stiffness ranges [3] and existence of optimal stiffness ranges for differentiated cell functions [4], [5], suggesting multiple levels of complexity in cell fate regulation by substrate stiffness.

We found that the effects of matrix stiffness on net fibroblast accumulation were consistent across a broad range of matrix densities and serum concentrations. While subtle variations in stiffness-dependent cell spreading have been reported with variations in matrix density [29], our results support a central and robust role for matrix stiffness in influencing net proliferation independent of underlying matrix ligand density. Whether such influences of stiffness remain robust across matrices of varying composition remain to be determined, though minor differences in cell responses to stiffness that depend on matrix presentation have been noted [30]–[32]. The methods described here for fabrication of multiwell stiffness-controlled plates are readily adaptable for conjugation of additional or alternative matrix components, and could serve as an efficient platform for further exploration on interactive effects of matrix ligand presentation and substrate stiffness on cell responses.

Our screen of small molecules, while limited in scope, revealed significant differences in growth modulation in more than half the compounds tested. We identified compounds with both increased and decreased potency on soft (1 kPa) relative to rigid substrates (glass), in addition to those with equivalent efficacy irrespective of stiffness. Interestingly, while targeting Rac with NSC23766 or focal adhesion kinase with FAK inhibitor 14 did not generate stiffness-dependent effects, targeting components of the cytoskeleton with taxol, a microtubule stabilizing agent, or cytochalasin D, a net actin depolymerizing agent, both resulted in much stronger inhibition of growth on rigid dishes, and relative ineffectiveness on soft substrates. In contrast, we identified two compounds, okadaic acid and cantharidin, that were more effective in growth attenuation on soft substrates than rigid dishes. Both are naturally-derived inhibitors of protein phosphatases 1 and 2A. Notably, the consistent stimulation of cell growth on soft substrates by blebbistatin, an effect not normally ascribed to this inhibitor of non-muscle myosin II, suggests that the actions of some compounds may be entirely obscured in rigid environments.

Together, these results provide a glimpse into the unexpected ways in which matrix stiffness may modulate cellular responses to biochemical factors. The potential for the discovery of molecules and pathways uniquely or differentially active within particular physiological stiffness contexts has significant implications for the development of new therapeutic strategies. Such efforts might be particularly fruitful when applied to the study of the cellular processes relevant to cancer, hypertension, and fibrosis [18], [33]–[36], all of which are accompanied by prominent changes in matrix stiffness.

## Materials and Methods

### Coverglass array assembly

Custom-cut borosilicate glass squares (Hausser Scientific) 0.2 mm smaller than the well dimensions (7.4×7.4 mm for 96 well plates, 2.8×2.8 mm for 384 well plates) were placed in each well of the corresponding multiwell plate to properly align them and affixed to 96 (Sigma) or 384 (Boekel) pin stainless steel replicators sanded to a flatness of ±10 µm for each pin. To render them hydrophobic, the glass array was treated with Surfasil (Thermo Scientific) according to the manufacturer's instructions. Prior to each casting run, the glass array was rinsed in methanol and dried using pressurized air.

### Multiwell gel casting

The wells of a glass-bottom, black or white-walled, 96 or 384 well plates (Matrical Biosciences) were filled with a 0.4% aqueous solution of 3-methacryloxypropyltrimethoxysilane (Acros Organics) at pH 3.5 for 1 hour, rinsed in distilled water and air dried. Solutions containing a final concentration of 0.12% ammonium persulfate, 0.15% tetramethylethylenediamine, 1 mM sodium bisulfate, and variable ratios of acrylamide:bisacrylamide (Bio-Rad) indicated in **Fig. 2A** were prepared by using a multichannel pipette to mix 1 part of a 10x solution of ammonium persulfate/sodium bisulfate/TEMED with 9 parts of acrylamide:bisacrylamide to give the desired final concentration. The solutions remained fluid for at least 30 seconds of mixing, providing sufficient time to deliver 5 µl of each pre-polymerization mixture to selected wells of a 96 well plate (1 µl for a 384 well plate); this was typically performed in a columnwise fashion. To create a thin PA gel layer, the glass array was fully inserted into the multiwell plate to sandwich the solutions, with small pieces of copy paper (∼60 µm thick) placed in the corner wells to control gel heights. After 10 minutes, the casting array was gently separated from the gels and removed. To derivatize the gels, 50 µl of sulfo-SANPAH (G-Biosciences) at 50 µg/ml in 50 mM HEPES buffer, pH 8.5 was delivered to each well and the crosslinker was activated by exposure to high intensity UV (GML High Output germicidal lamp) for 2 minutes. The solution was replaced with 100 µl of bovine collagen (PureCol) in PBS at 10 µg/ml (unless otherwise indicated) and incubated for a minimum of 2 hours at room temperature. The gels were exposed to UV for 1 hour prior to all validation and cell culture studies.

### Automated fluorescence microscopy

Cells in 384 well plates were imaged using a Pathway HT (AttoBioscience) fluorescence imaging system with autofocusing (Vollath F4 algorithm) set at a 400 nm step stage positioning. For measurements of gel thickness, the system was used to measure the relative z-positions of 1 µm fluorescent YG carboxylate microspheres (Polysciences) bound to glass surface of a 96 well glass-bottom plate and on the surface of PA gels representing each stiffness condition.

### Hydrogel stiffness measurements

PA gels formed within a 96 well plate were removed by punching out the glass bottom of individual wells with forceps. The gels were mechanically characterized using an Asylum MFP-3D atomic force microscope. Force-indentation profiles were acquired at an indentation rate of 20 µm/second using a sphere-tipped probe (Novascan) with a diameter of 5 µM and a nominal spring constant of ∼60 pN/nm. Young's modulus was calculated from fitting force-indentation data using a Hertz sphere model.

### Collagen density measurements

Gels conjugated with various amounts of collagen were blocked with 1% goat serum in PBS for 1 hour, and incubated for 2 hours with a mouse monoclonal antibody against native type I collagen (COL-1, Sigma) diluted 1∶250 in PBS. Gels were washed 3x with 0.1% Triton X-100 in PBS for 5 minutes each, and incubated for 1 hour with a goat anti-mouse HRP-conjugated antibody (Cell Signaling) diluted 1∶1000 in PBS. The gels were washed 3x for 15 minutes each, and then 100 ul of Supersignal West Pico Chemiluminescence Substrate (Pierce) was added to each well. Images were captured using a CCD camera (Syngene) within a linear range of detection, and average pixel densities were evaluated in Adobe Photoshop 6.0.

### Cell culture and assays

For all experiments, normal human lung fibroblasts (Lonza) were used at passage 3-6. Human bone marrow-derived mesenchymal stem cells (Tulane University) were used at passage 1–3, and all other cell types were obtained from ATCC. All cells were cultured in Kaighn's Modification of Ham's F12 Medium (F12K) supplemented with 10% fetal bovine serum, 100 U/ml penicillin and 100 µg/ml streptomycin (all from Mediatech) in a humidified 37°C incubator with 5% CO_2_. For multiwell plate assays, the top and bottom rows served as cell-free background controls for each stiffness context represented. Relative cell numbers were assessed by the Cyquant NF Cell Proliferation Assay (Invitrogen). To directly evaluate proliferation, relative amounts of incorporated bromodeoxyuridine (BrdU) were determined using a colorimetric, Cell Proliferation ELISA (Roche Applied Science) following a 24 hour exposure to BrdU. Apoptosis was assessed using a fluorescence-based, ApoONE Caspase 3/7 Activity Assay (Promega).

### Cell growth profiling

Cells were seeded in serum-free F12K media at <50 cells/mm^2^ in multiwell plates specifying five stiffness contexts, and allowed to attach for 4 hours. Media was replaced with F12K containing 10% serum and cells were cultured for an additional 72 hours. For each stiffness condition, fold change was expressed as the ratio of adherent cell number at 72 versus 4 hours.

### Drug screening

GF109203X, NSC23766, simvastatin, PP1, FAK inhibitor 14, SU9516, okadaic acid, cantharidin, taxol, IPA3, cytochalasin D, (+/−) blebbistatin, and calpeptin were purchased from Tocris Bioscience; dexamethasone and PD173074 from Sigma; SP600125, cycloheximide and ML-7 from Calbiochem. Cell numbers were evaluated 72 hours after a single addition of the indicated concentrations of drugs, which were applied 4 hours after cell seeding.
